# Turtles All the Way Down: From *g* to Mitochondrial Functioning †

**DOI:** 10.3390/jintelligence8020023

**Published:** 2020-05-21

**Authors:** Lazar Stankov

**Affiliations:** School of Psychology, The University of Sydney, Sydney, NSW 2006, Australia; lazondi@rocketmail.com

## 1. Turtles All the Way Down: From *g* to Mitochondrial Functioning

Geary (2018, 2019) theorizes that the efficiency of mitochondrial functioning is the fundamental biological mechanism that affects the organism as a whole and is common to all brain and cognitive processes. This is a plausible suggestion—cellular metabolic processes should affect both physical and psychological functioning of a biological system. The evidence that was reviewed in support of this line of theorizing is extensive, and the theory is likely to have a considerable impact on the study of intelligence.

However, I feel that there are several issues that need to be discussed further. These are mostly related to psychometrics rather than cellular metabolism.

## 2. *g* Is Weak: We Need to Focus on Gf, Gc, and the Other Broad Abilities

Geary (2018) starts by pointing to the importance of *g* and states that the first principal component is strong, since it captures a large amount (50–60%) of common variance in studies based on batteries of tests of cognitive abilities. However, it is well known that the percentage of common variance captured by the first principal component depends on the nature of the tests included in the battery—i.e., it depends on how we define what Spearman referred to as “all branches of intellectual activity”. If this is understood as what we refer to today as the totality of cognitive processes, the average correlation of r = 29 reported by Carroll in his 1993 survey of human cognitive abilities leads to the conclusion that only about 35% of the common variance is accounted for by the first principal component. In other words, *g* estimated from such a correlational matrix will have to be weaker than is cited in the target article. Furthermore, when test batteries include complex cognitive tasks that do not tap mental processes captured by contemporary IQ tests—e.g., non-trivial tasks linked to different sensory modalities, speed of cognitive processing, and decision-making and rationality—the percentage accounted for by the first principal component declines to around 25%, or approximately half of what Geary has stated (Stankov 2001). A similar value (25.9%) for the first factor was obtained with a battery of 25 tests, consisting of 12 measures of fluid and crystallized intelligence, 6 measures of mental speed, and 7 tactile and motor tasks, including the Halstead–Reitan Neuropsychological Test Battery, which was presumed to measure “biological intelligence” (Pallier et al. 2000).

If *g* is not as strong as some scholars have assumed, it may be profitable to focus on the broad abilities at the lower levels in the hierarchy. Fluid (Gf) and crystallized (Gc) intelligence come to mind but several other factors occupy the same status in the Cattell–Horn–Carroll (CHC) theory (Schneider and McGrew 2018). Assuming the presence of a strong *g*, it can be expected that all broad factors will be affected by the mitochondrial efficiency to some extent. However, in reality, *g* is simply not as strong as Geary (2018, 2019) has assumed.

It is also possible, as some extant studies show, that Gf is the main factor that is sensitive to mitochondrial functioning. Gc is likely to be less affected by the biological processes related to health and development (aging) than is Gf. Additionally, abilities related to sensory modalities (e.g., broad visualization [Gv] and broad auditory function [Ga]) may behave like Gf, while long-term storage and retrieval (Glr) ability may be similar to Gc. When rotated, some of these broad abilities, or combinations thereof, capture an amount of variance similar in size to that of *g* itself.

A recently proposed process-overlap theory (POT) (Kovacs and Conway 2019) both denies the existence of *g* in the form described in the target article and acknowledges the relevance of abilities identified by the CHC theory. POT points out that *g* should be viewed as a formative construct rather than an explanatory factor and therefore makes the existence of a single biological process less likely. Geary (2018) is aware of the CHC theory and POT’s account of *g* but chooses to ignore them in favor of a single *g*.

I can accept that, although the general factor is relatively weak, mitochondrial efficiency may still be at its core and may therefore be seen as the most fundamental underlying biological mechanism affecting individual differences in a broad range of cognitive abilities. In other words, the relevance of the broad factors below the highest-order general factor will not disconfirm the mitochondrial hypothesis but merely diminish its role as the dominant biological mechanism. Each broad factor in the CHC theory may have a different biological (or other) underlying mechanism in addition to the mitochondrial function. Thus, biological processes at the inter- and intra-modular levels and perhaps also at the neural and glia level in Geary’s model would increase in importance.

## 3. Mitochondrial Efficiency and *g*: Additional Evidence Is Needed

But what do we know about the (causal?) relationship between neuroenergetics and cognitive abilities captured by tests of intelligence? The existing evidence, in my opinion, is not yet sufficiently convincing.

First of all, it is important to keep in mind that the identification of the *g* factor was based on studies of people who live healthy lives in a community. Studies pointing to the effects of mitochondrial functioning, however, have focused on nonhuman animals and on the high energy demands of the human brain. A review paper by Killeen et al. (2016) is cited by Geary (2018) in support of the argument that the efficiency of mitochondrial functioning contributes to individual differences in human cognitive abilities. Some of the reviewed studies do support such a claim, but several of them also employ reaction time measures rather than accuracy scores commonly used in IQ tests, and often study performance under conditions of fatigue or stress. These measures and treatment conditions are not typical of those used in individual differences research and, therefore, the studies do not provide unequivocal support for the view that individual differences in mitochondrial functioning underlie individual differences captured by *g*.

Some other findings are not supportive of that link. For example, my quick search of recent literature identified a study (Shurtleff et al. 2018) based on children (N = 49) affected by mitochondrial disease, which supports the conclusion that some diagnosed patients may indeed have impaired intelligence. Twenty-four of these 49 patients had epileptic seizures. IQ scores were available for all participants. The seizures group was found to have a median IQ score of 67, whereas the median score of the group without seizures was exactly on the population average IQ of 100. This leads to the conclusion that mitochondrial disease can indeed lead to an impaired IQ, but this occurs only in those patients who have epileptic attacks as part of the syndrome. Based on this one (admittedly small) study, mitochondrial disease on its own does not seem to impair cognitive function. This too supports the need for further examination of the notion that mitochondrial functioning underlies individual differences in intelligence.

Theorizing about the relationship between *g* and mitochondrial functioning is also linked to the effects of health and aging, which are cited as providing auxiliary evidence for the main arguments about the biological bases of *g.* Since our team has done work in both of these areas in the past, I will mention my concerns about this evidence as well.

## 4. Health and Intelligence: All-Cause Mortality, Cardiovascular Problems and Diabetes

Clearly, from the example above (Shurtleff et al. 2018), it seems necessary to study in more detail the IQ of patients directly affected by mitochondrial disease. However, at least some measures of health mentioned in Geary’s (2018) review do not tap mitochondrial function directly but are linked to it through certain mediating pathways.

As pointed out by Geary (2018), the relatively new field of cognitive epidemiology was preceded by work showing that the intelligence scores of Australian soldiers who returned from the Vietnam war predicted their mortality within ten years of discharge from the military. There were two main causes of death in that sample: suicides and motor vehicle accidents (O’Toole and Stankov 1992). Similar findings related to mortality and *g* were subsequently obtained using several broad health-related measures (Geary 2018, 2019), and it is now accepted that there is a correlation between cognitive abilities and all-cause mortality. One specific factor, cardiovascular health, is important since it has been linked to mitochondrial functioning and intelligence (Geary 2018). Such a link will need to be confirmed for several other measures of health status and, of course, it will be hard to show a direct link for some causes of mortality (e.g., motor vehicle accidents).

Diabetes is another disease that has been linked to both efficient cerebral metabolic processes and cognitive abilities. Early interpretations of Spearman’s *g* as mental energy were in terms of capacity (individual differences in intelligence arise as a consequence of a different number of functionally active neurons). In the late 1980s, efficiency (individual differences in intelligence arise from the same number of neurons that differ among themselves with respect to their functional efficiency) was proposed. It was also observed that cognitive changes that accompany the time course of diabetes are similar to those observed in aging. A review of the available literature at the time did show that Gf was correlated with functional efficiency, assessed using measures of cerebral metabolic rate for glucose (CMRgl), and task complexity proved to be an important aspect of the relationship (Stankov and Dunn 1993).

With regards to health, it will be important to identify diseases that are affected, directly or indirectly, by mitochondrial functioning and demonstrate their link to measures of *g*. Further studies of mitochondrial disease, cancer, and diabetes in relation to intelligence are also needed. In my opinion, the “body integrity hypothesis” (Deary 2008) is simply too vague.

## 5. Aging and Intelligence: Mixed Findings

An often-cited finding in the life development literature is that *g* is strong (i.e., captures a larger percentage of common variance) among children, differentiates into Gf, Gc and other factors past the teenage years and adulthood, and becomes dedifferentiated again among the elderly (say, over 60 years of age). Recent evidence based on a meta-analysis of the longitudinal data also indicates that there is a single factor that underlies individual differences in cognitive change, providing support for what has been labeled the “dynamic” dedifferentiation hypothesis. That cognitive change factor and the estimate of *g* are moderately correlated (r = 0.49) (Tucker-Drob et al. 2019). This finding is used to make an important point, namely, that there is a common mechanism (such as mitochondrial processes) that supports cognition, and which declines as a natural consequence of biological aging (Geary 2019).

Our own past studies involving measures that were labeled as indicators of primary aging (tests of sensory and motor functions) and secondary aging (Gf, Gv and Ga) among the elderly did support the dedifferentiation hypothesis and led us to conclude that the decline in Gf is related to biological changes in the brain, central nervous system, and motor system (Anstey et al. 1993, p. 568). Our work, however, was based on the older, “static” rather than “dynamic”, version of the dedifferentiation hypothesis. Because of the scarcity of longitudinal studies, individual change scores were rarely available in the past. Most life-span developmental work relied on the cross-sectional design and the higher *g* among the samples of elderly participants obtained with this design is today referred to as “static” dedifferentiation. I mention this because recent investigations of the static dedifferentiation hypothesis have produced mixed results. For example, a study based on the standardization sample for the Woodcock–Johnson IV Intelligence Test Battery ranging in age from 25 to over 80 found that the correlation between Gf and Gc (r = 0.65) remained invariant with respect to age (Hartung et al. 2018). In other words, *g* is not stronger in samples of older participants. This finding was also confirmed when the static dedifferentiation hypothesis was examined by Tucker-Drob et al. (2019).

In summary, support for the traditional “static” version of the dedifferentiation hypothesis has come into question recently. Since Geary’s claims about the common (i.e., mitochondrial) processes in *g* and aging are based on the “dynamic” (longitudinal) version of the dedifferentiation hypothesis, I feel that it may be sensible to wait for additional evidence before its full acceptance. Therefore, strong claims that aging data support the proposition that mitochondrial processes are the linchpin of general intelligence seem premature.

## 6. Moving Forward

It is essential to develop an agreed upon and reliable measure of neuro-energetic processing. I suspect that this measure would have to be relevant to brain functions rather than to other organs and, perhaps, even to cellular activity within particular parts of the brain. For example, motor neuron disease seems to have affected Stephen Hawking’s intelligence little, if at all.

Once developed, such a measure can be linked to the “myriad theories and research traditions” (Geary 2018) that have been proposed and tested in a search for the basis of *g*. A few of these did not rely on speculation but on empirical evidence and cannot be easily dismissed, especially if they relate to some of the lower-order factors. For example, Gamma-band oscillations obtained from EEG recordings that can be classified as indicators of intramodular processes in Geary’s (Geary 2018, 2019) model have been interpreted as a mechanism of “network binding” and implicated in various aspects of perception, memory, and cognition. Moderate-sized correlations were obtained between scores derived from a battery of fluid and crystallized intelligence tests and magnitude and latency indices of Gamma synchrony from different sites (Stankov et al. 2006). Although it is plausible to assume that mitochondrial functioning affects the efficiency of phase-synchronization, I am not aware of any empirical support for such a claim.

A good measure of cellular energy production will need to be shown to correlate directly with measures of *g.* Ideally, proof of a causal link would be needed.

## 7. All Turtles, All the Way Down, Are Important

The nested model presented graphically in Geary (2018, 2019) and described as implying that the “deficits or inefficiencies at lower levels will ripple through all higher levels” is welcome, since it is in the spirit of an old argument that reductionistic explanations of *g* should be in terms of biological processes, not in terms of presumably elementary cognitive tasks (ECTs) (Stankov 2005).

Most of the critical points discussed above can be accounted for or adopted within the model. However, the shift of emphasis from *g* to cognitive processes at the lower order in the CHC theory complicates the picture significantly, since these broad abilities can be expected to rely on the intermediate processes nested between the inner and outer circles of the figure in Geary (2018, 2019), not only those on the bottom level. The relative strength of the relationship between mitochondrial efficiency and higher levels of processing may vary from one broad factor to the other. There are also questions about the interplay between the middle levels—for example, do the ripples go in both directions, or only up? Another question that comes to mind is: is there is a role for neural plasticity in the model, and does it apply to mitochondrial function?

In short, the prospect of having each broad factor affected by different combinations of intra-modular, inter-modular or lower level mitochondrial processes is real, and the overall picture is likely to be more complicated than suggested by an infinite regress model, which is as it should be.
